# Listening to patients: using verbal data in the validation of the Aberdeen Measures of Impairment, Activity Limitation and Participation Restriction (Ab-IAP)

**DOI:** 10.1186/1471-2474-11-182

**Published:** 2010-08-12

**Authors:** Jeremy Horwood, Beth Pollard, Salma Ayis, Teresa McIlvenna, Marie Johnston

**Affiliations:** 1 Department of Community Based Medicine and Clinical Science at North Bristol, University of Bristol, UK; 2 College of Life Sciences and Medicine, University of Aberdeen, UK; 3 Department of Public Health Sciences, King's College London, UK; 4 University Hospitals Bristol NHS Foundation Trust, UK

## Abstract

**Background:**

The purpose of the study was to evaluate the validity of the self-administered Aberdeen Measures of Impairment, Activity Limitation and Participation Restriction (Ab-IAP): by investigating how participants interpret and respond to questions using the cognitive interviewing technique.

**Methods:**

Twenty patients with osteoarthritis of the knee or hip participated in a cognitive interview whilst completing the Ab-IAP. Interviews were conducted using the concurrent 'think aloud' design. All interviews were audio recorded and transcribed verbatim and analysed (i) using a standardised classification scheme to identify four types of response problems and (ii) thematically using the constant comparative technique.

**Results:**

Participants used various response strategies when answering questions about impairment, activity limitations and participation restriction. Problems were judged to be present in 3.1% of participants' responses for the item Ab-IAP_. _Thematic analysis provided insight into the type and nature of problems people experienced when completing the Ab-IAP measures. The problems identified were mainly comprehension and response problems.

**Conclusions:**

Participants had minimal difficulties completing the Ab-IAP; however those difficulties identified have prompted suggestions for improving the measures. The cognitive interviews produced results that were compatible with statistical analysis of the measures.. Cognitive interviewing was beneficial for testing the validity and acceptability of new Ab-IAP measures. The results demonstrates that the Ab-IAP, in addition to being theoretically-based and having good psychometric properties, elicits appropriate responses.

## Background

The Aberdeen Measures of Impairment, Activity Limitation and Participation Restriction (Ab-IAP [1] were developed to reflect the International Classification of Functioning, Disability and Health (ICF) definitions of these three components [2]. The measures were developed for people with hip and knee osteoarthritis. As it has been shown that existing osteoarthritis measures mixed up these components [3], the Ab-IAP was specifically developed to reflect each component as accurately as possible without contamination from the other constructs within the ICF model. The items in the Ab-IAP were based on items from 13 existing osteoarthritis measures that had been judged to be only measuring a unique ICF construct [3]. A statistical item analysis was previously carried out on the pool of 59 unique items using both classical test theory and item response theory [1]. The resultant 35-item Ab-IAP was shown to have good psychometric properties [1], however further validation studies were needed as the validation of any measure is an additive process. Having developed the Ab-IAP to truly reflect the components of the ICF theoretical framework, it was important to ascertain whether respondents completing the Ab-IAP interpreted the items as they were designed to be interpreted. Hence, the primary aim of the study was to validate the 35 item Ab-IAP with the results being used to inform future revisions to the Ab-IAP measures. The secondary aim was to compare whether items that people have difficulties interpreting corresponded to items identified by the previously reported item analysis (i.e. the analysis that reduced the pool of items from 59 to the 35 in the current version of the Ab-IAP).

When developing a measure, it is key that researchers examine how the items are understood from the participants' perspective to identify potential response problems that may arise through misunderstandings, ambiguous concepts, inconsistent interpretations and context effects of items. Cognitive interviewing techniques were developed as a means of gaining participant feedback to help researchers create more user-friendly measures [4]. By examining how participants interpret self-completion measures, improvements can be made that reduce the number of unanswered questions and response errors, and raise overall response rates [5,6]. One of the main techniques of cognitive interviewing is 'think aloud' interviewing. In 'think aloud' interviews [7], participants are asked to 'think aloud' as they answer survey questions [8], thus verbalizing the thoughts that would normally remain silent. Participants are not asked to explain or justify what they are doing and they are not asked to report their strategies. The researcher records these verbalizations, which are then transcribed verbatim and subjected to analysis. A review of this methodology generally indicated that the verbalization of ongoing thoughts as it happens without elaboration or explanation has no significant effect on the quality of the performance of the task, other than some slowing of the task [7]. The method manages to avoid altering the interviewee dynamic in any significant way, which might affect the study's comparability with 'normal' usage of the measure [9].

The methodology can be useful in identifying problematic items that can then be amended before use in the field [10]. 'Think aloud' methodology has been shown to be appropriate for developing, refining or evaluating/validating measures on a range of health care issues [9,11-14]. The 'think aloud' technique can provide a useful method for improving the acceptability and validity of research instruments in health research applications [10].

This paper reports the use of the 'think aloud' technique in evaluating the Ab-IAP. The context for the study is in people with hip and knee osteoarthritis. The paper provides both quantitative and qualitative assessments of how participants interpreted and responded to the Ab-IAP.

## Method

### Design

Concurrent think aloud design was used in this study. The participants were asked to 'think aloud' and verbalise his/her thought process as they competed the items.

### Participants

The sample was patients (*n *= 20) with confirmed diagnosis of osteoarthritis of the knee or hip. This population was selected as the Ab-IAP measures were developed for people with hip and knee osteoarthritis. Participants were recruited from either a pre-operative assessment clinics or at their one-year follow up appointment at orthopaedic outpatient clinics at two NHS trusts. Five participants from each of the following groups were recruited (1) pre-operative primary knee replacement surgery patients, (2) one-year post-operative primary knee replacement surgery patients, (3) pre-operative primary hip replacement surgery patients and (4) one-year post-operative primary hip replacement surgery patients. Participants were purposively selected for a mix of social class, education, age and gender. Participants were excluded if they had a diagnosis of dementia, were unable to give informed consent or had a poor understanding of English language. The study was approved by the Local NHS Research Ethics Committee and NHS Research and Development office and research governance arrangements were followed.

### Instruments

The 59-items presented to the participants were from the initial pool of items that had been previously identified as measuring only a single ICF construct [3] i.e. only impairment or activity limitation or participation restriction (13 Impairment, 26 Activity limitation, 20 Participation restriction items) [1]. A statistical item analysis, combining classical test theory and item response theory, on this pool of 59 items has been reported elsewhere [1] and resulted in a subset of 35-items that formed the the Ab-IAP (9 Impairment, 17 Activity limitation and 9 Participation restriction items)[1]. Participants answered each item by choosing one of five response options.

Participants were additionally asked to also complete a measure covering socio-demographic characteristics, pain scores and details of their joint replacement surgery.

### Procedure

Participants took part in the think aloud task in their own homes or in a private room at the clinics, according to the participants preference. Full written consent was obtained from participants before proceeding with the study.

To ensure each participant was comfortable with the process and understood what was required, they were asked to 'think aloud' three practice items. Any queries or problems were dealt with at this stage by the researcher. The researcher then sat out of the line of sight of the participant. Once participants began completing the measures, they were not interrupted, unless the participants paused for longer than 10 seconds, in which case the researcher quietly reminded the participant to "keep thinking aloud". All other interactions between the participant and the interviewer were kept to a minimum so as not to interfere with the participant's completion of the measures. This approach was adopted to try and avoid altering the way participants answered the measures to make the study comparable to normal usage of the measures.

Each 'think aloud' session was digitally audio recorded and transcribed verbatim. JH and TM facilitated the 'think aloud' sessions and collected all data, after each completing three pilot 'think aloud' interviews and discussing the procedure.

### Analysis

The interview transcripts were first analysed for problems in the participants' undertaking of the task. The first two authors independently examined the transcripts, to segment them into material relating to each of the 59 items from the pool of Ab-IAP items. Item-by-item analysis was then performed on the written texts independently by the authors (JH, BP) in relation to the participant's questionnaire scores, identifying where and how the items failed to achieve its measurement purpose.

A standardised classification scheme was employed to identify four types of response problems and the distribution of these problems. The classification system was employed to increase consistency in the scoring of the transcripts and to allow for standardisation of the process of interview analysis. The classification scheme employed was based on the 'question and answer' model, developed in cognitive psychology and is the background theory underlying cognitive interviewing [8]. The model suggests that participants perform four actions when completing a measure in order to answer an item [15] and problems can occur at each stage and stages being interconnected. The four stages are; (1) comprehension (e.g. any misunderstanding of a word, phrase, or response option), (2) retrieval (e.g. a recall problem or a miscalculation of the time frame stated in the item), (3) judgment (e.g. the participants response does not match that of the investigators intent for the item or the recalled experiences are irrelevant or inadequate) and (4) response (e.g. participants response is inconsistent with the personal experience expressed or the desired response is missing from the response choices). A score was made for each item, by summing problems for these four categories. It was additionally noted when the participants 'struggled' to answer an item (e.g. rereading the item several times, or questioning how sensible the item was), even when they finally arrived at a correct response. It was also noted when the participants felt there was 'insufficient information' in the item for it to be answered (e.g. when it is not clear what question the item is asking).

In addition to the quantitative analysis, a thematic analysis of the transcripts was conducted independently. The transcripts were imported into the software package Atlas.ti [16], and a thematic analysis of the findings was undertaken using the constant comparative techniques in which themes and codes were compared within and across transcripts to refine understanding of the emerging results [17]. Transcripts were read and re-read for meaning and understanding and inductive codes assigned to segments of data that provided insight into the type and nature of problems participants experienced completing the Ab-IAP. Descriptive accounts were generated which successively incorporated each new transcript until a full account was obtained.

## Results

The twenty participants were aged between 32 and 86 (mean 71 years SD 12). Nine of the participants were men and eleven were female. All twenty of the participants classified their ethnicity as white. Fifteen participants were educated to O'level, four attended further education and one had a university degrees. Six participants had a social class of managerial and technical, seven non-manual skilled occupations, three manual skilled occupations and four partly skilled occupations [18]. One participant was single, thirteen married or in a relationship, one divorced or separated and five widowed. Eight participants lived alone. Eighteen participants were retired. Pre-operative participants took part in the study 1 to 32 days (mean 14 days) before their operation. Post-operative participants took part in the study 9 to 19 months after their operation (average 13 months). The task took participants between 15-52 minutes to complete (average 32 minutes).

### Distribution of judged problems

Between zero and twenty problematic segments per participants were judged to be present. As Table 1 illustrates, fifteen participants were judged as having problems completing the measures using the four classifications of problems, a further three participants struggled but answered the measures correctly and two participants had no problems completing the measures. The majority of problems that occurred were comprehension or response problems (although the majority of response problems were from one pre-operative knee participant). This was mostly due to participants ticking more than one response option due to their arthritis being highly variable (as illustrated in the qualitative analysis) and also contributed to more struggles being identified within the pre-operative knee group. Ten participants felt that items had insufficient information for them to be easily answered.

**Table 1:** Frequency and type of agreed judged problematic segments for the twenty participants completing the Ab-IAP (59-item).

Participant characteristics						Judged problems^2^							
						
No.	M/F^1^	Age	Hip/Knee Joint replacement	Pre/Post Surgery	Number of items with no identified problems	C	R	J	Rp	Total problems	Struggled	Struggled but answered item correctly	Insufficient information
P1	M	79	Hip	Pre	59	0	0	0	0	0	0	0	1

P3	F	82	Hip	Pre	58	1	0	0	0	1	6	5	0

P9	F	32	Hip	Pre	59	0	0	0	0	0	7	6	0

P11	F	69	Hip	Pre	57	3	0	0	0	3	4	1	1

P13	F	80	Hip	Pre	54	0	0	0	5	5	3	2	0

P20	F	75	Knee	Pre	55	2	0	0	2	4	7	7	2

P4	M	81	Knee	Pre	58	1	0	0	0	1	4	3	3

P5	F	69	Knee	Pre	39	4	0	0	16	20	9	2	4

P6	M	86	Knee	Pre	56	0	0	1	2	3	1	0	0

P19	F	74	Knee	Pre	59	0	0	0	0	0	3	3	0

P8	F	73	Hip	Post	54	0	0	4	0	4	2	0	1

P10	M	74	Hip	Post	59	0	0	0	0	0	1	1	0

P16	F	69	Hip	Post	57	0	0	0	2	2	3	3	1

P17	M	70	Hip	Post	58	1	0	0	0	1	7	6	2

P18	M	70	Hip	Post	56	2	0	1	0	3	0	0	0

P2	F	53	Knee	Post	57	2	0	0	0	2	5	5	5

P7	M	75	Knee	Post	58	1	0	0	0	1	0	0	0

P12	M	63	Knee	Post	58	1	0	0	0	1	5	5	0

P14	F	61	Knee	Post	55	3	0	0	1	4	4	1	3

P15	M	77	Knee	Post	59	0	0	0	0	0	0	0	0

Total					1125	21	0	6	28	55	71	50	23

^1 ^M = male and F = female. ^2 ^C = Comprehension, R = Retrieval, J = Judgement, Rp = Response

The frequency of problematic segments for each of the 59 items demonstrates that between zero and ten problems were judged to be present for each item (Tables 2, 3, 4 and 5). No retrieval problems were identified, this may be due to none of the items asking participants to recall details of frequency of events, but also suggests that asking the participants to recall their experiences over the past four weeks was an achievable task. The least proportion of total problems were judged to be present for the impairment construct items, however the highest proportion of struggles were identified within this construct (Table 2). The majority of response problems were judged to be present within the activity limitation construct (Table 3). The participation restriction items yielded the highest proportion of total problems and the most comprehension problems (Table 4), with item C14 'How healthy is your physical environment?' being identified as the most problematic item of the measures (this item being dropped in the Ab-IAP measures). Out of the 1180 segments that were analysed from the pool of 59 items, problems were identified in 4.7% (Table 5). Problems were identified in 3.1% of the 700 segments that were analysed for the 35-item Ab-IAP (Table 6.).

**Table 2:** Frequency and type of agreed judged problematic segments for Impairment items of the Ab-IAP (59-item).

Item	Number of participants with no identified problems	Judged problems^1^				Total problems	Struggled	Struggled but answered item correctly	Insufficient information
						
		C	R	J	Rp				
A01 How would you describe the pain you usually have from your joint?	19	0	0	0	1	1	5	4	1

A02 How often have you had severe pain from your arthritis?	18	1	0	0	1	2	2	1	2

A03* How often have you had pain in two or more joints at the same time?	20	0	0	0	0	0	1	1	0

A04 Does remaining standing for 30 minutes increase your pain?	20	0	0	0	0	0	3	3	1

A05 How active has your arthritis been?	18	2	0	0	0	2	6	4	2

A06 Have you been troubled by pain from your joint in bed at night?	20	0	0	0	0	0	0	0	0

A07 How long has your morning stiffness usually lasted from the time you wake up?	20	0	0	0	0	0	2	2	0

A08* Has pain from your joint kept you awake during your night-time sleep?	20	0	0	0	0	0	0	0	0

A9 Have you had any sudden, severe pain - 'shooting', 'stabbing' or 'spasms' - from the affected joint?	20	0	0	0	0	0	0	0	0

A10 Have you felt that your knee or hip might suddenly "give way" or let you down?	20	0	0	0	0	0	1	1	0

A11* What degree of difficulty do you have bending and rotating your affected joint?	20	0	0	0	0	0	1	1	0

A12 How severe is your stiffness after first wakening in the morning?	19	0	0	0	1	1	1	1	0

A13* How severe is your stiffness after sitting, lying or resting later in the day?	17	1	0	1	1	3	0	0	0

Total	251	4	0	1	4	9	22	18	6

^1 ^C = Comprehension, R = Retrieval, J = Judgement, Rp = Response. * Items removed to form the Ab-IAP (35 item).

**Table 3:** Frequency and type of agreed judged problematic segments for Activity Limitation items of the Ab-IAP (59-item).

Item	Number of participants with no identified problems	Judged problems^1^				Total problems	Struggled	Struggled but answered item correctly	Insufficient information
						
		C	R	J	Rp				
B01* What degree of difficulty do you have descending stairs?	20	0	0	0	0	0	1	1	1

B02* What degree of difficulty do you have ascending stairs?	20	0	0	0	0	0	1	1	1

B03 What degree of difficulty do you have rising from sitting?	19	0	0	0	1	1	1	1	0

B04* What degree of difficulty do you have standing?	20	0	0	0	0	0	1	1	0

B05 What degree of difficulty do you have bending to floor?	19	0	0	0	1	1	1	0	3

B06 What degree of difficulty do you have walking on flat?	19	0	0	0	1	1	2	2	0

B07* What degree of difficulty do you have putting on socks/stockings?	17	0	0	0	3	3	1	0	0

B08 What degree of difficulty do you have rising from bed?	19	0	0	0	1	1	0	0	0

B09 What degree of difficulty do you have taking off socks/stockings?	18	0	0	0	2	2	0	0	0

B10 What degree of difficulty do you have lying in bed?	20	0	0	0	0	0	2	2	0

B11 What degree of difficulty do you have sitting?	19	0	0	1	0	1	2	2	0

B12 What degree of difficulty do you have getting on/off toilet?	20	0	0	0	0	0	0	0	0

B13* What degree of difficulty do you have walking long distances on flat (> 1/2 mile)?	18	1	0	0	1	2	2	1	0

B14 What degree of difficulty do you have climbing up and down one flight of stairs?	19	0	0	0	1	1	0	0	0

B15 What degree of difficulty do you have climbing up and down several flights of stairs?	19	0	0	0	1	1	1	1	0

B16 What degree of difficulty do you have dressing yourself (except shoes and socks)?	20	0	0	0	0	0	0	0	0

B17 What degree of difficulty do you have putting on/off shoes?	20	0	0	0	0	0	0	0	1

B18 What degree of difficulty do you have washing and drying yourself?	20	0	0	0	0	0	1	0	0

B19 What degree of difficulty do you have washing your hair?	20	0	0	0	0	0	0	0	0

B20 What degree of difficulty do you have lifting?	19	0	0	0	1	1	5	4	4

B21* What degree of difficulty do you have kneeling?	20	0	0	0	0	0	0	0	0

B22* What degree of difficulty do you have walking short distances on the flat (100 yards)?	19	0	0	0	1	1	1	0	0

B23* Do you use a walking stick?	19	0	0	0	1	1	2	1	0

B24 Do you need someone to help you when you are walking?	19	0	0	0	1	1	0	0	0

B25 Do you need someone to help you go upstairs?	19	0	0	0	1	1	1	1	0

B26* Do you need someone to help you go downstairs?	19	0	0	0	1	1	0	0	0

Total	500	1	0	1	18	20	25	18	10

^1 ^C = Comprehension, R = Retrieval, J = Judgement, Rp = Response. * Items removed to form the Ab-IAP (35 item).

**Table 4:** Frequency and type of agreed problematic judged segments for Participation Restriction items for the Ab-IAP (59-item).

Item	Number of participants with no identified problems	Judged problems ^1^				Total problems	Struggled	Struggled but answered item correctly	Insufficient information
						
		C	R	J	Rp				
C01 How does your joint problem restrict you getting on with people (friends and family)?	20	0	0	0	0	0	1	1	0

C02 How does your joint problem restrict you having friends or relatives over to your home?	20	0	0	0	0	0	1	1	0

C03 How does your joint problem restrict you visiting friends or relatives?	19	0	0	0	1	1	1	1	0

C04 How does your joint problem restrict you telephoning friends or relatives?	20	0	0	0	0	0	0	0	0

C05 How does your joint problem restrict you showing affection?	18	1	0	1	0	2	0	0	0

C06 How does your joint problem restrict you doing your usual social activities?	20	0	0	0	0	0	1	1	0

C07 How does your joint problem restrict your opportunities for leisure activities?	20	0	0	0	0	0	1	1	0

C08* How does your joint problem restrict you doing your hobbies?	20	0	0	0	0	0	0	0	0

C09* How does your joint problem restrict how much money you have?	18	1	0	1	0	2	1	0	0

C10 How does your joint problem restrict you affording things you need?	20	0	0	0	0	0	0	0	0

C11* How does your joint problem restrict your capacity for work?	19	0	0	1	0	1	0	0	0

C12* How does your joint problem restrict your use of transport?	19	0	0	0	1	1	2	2	1

C13* How much do you enjoy life?	20	0	0	0	0	0	1	1	0

C14* How healthy is your physical environment?	10	9	0	0	1	10	8	2	3

C15* How available to you is the information that you need in your day-to-day life?	14	4	0	0	2	6	2	1	1

C16* How satisfied are you with your personal relationship?	17	1	0	1	1	3	4	2	2

C17* How satisfied are you with the support you get from your friends?	20	0	0	0	0	0	1	1	0

C18* How satisfied are you with the conditions of your living place	20	0	0	0	0	0	0	0	0

C19* How satisfied are you with your access to health services?	20	0	0	0	0	0	0	0	0

C20 How much of the time has your physical health or emotional problems interfered with your social activities (like visting friends, relatives etc.) ?	20	0	0	0	0	0	0	0	0

Total	374	16	0	4	6	26	24	14	7

^1 ^C = Comprehension, R = Retrieval, J = Judgement, Rp = Response. * Items removed to form the Ab-IAP (35 item).

**Table 5:** Frequency and percentage (%) of agreed judged problematic segments for the Ab-IAP (59-item).

Items	Number of items with no identified problems	Judged problems				Total problems	Struggled	Struggled but answered item correctly	Insufficient information
						
		Comprehension	Retrieval	Judgement	Response				
Impairment (13 items)	251 (96.5)	4 (1.5)	0 (0)	1 (0.4)	4 (1.5)	9 (3.5)	22 (8.5)	18 (6.9)	6 (2.3)

Activity Limitation (26 items)	500 (96.2)	1 (0.2)	0 (0)	1 (0.2)	18 (3.5)	20 (3.8)	25 (4.8)	18 (3.5)	10 (1.9)

Participation Restriction (20 items)	374 (93.5)	16 (4)	0 (0)	4 (1.0)	6 (1.5)	26 (6.5)	24 (6.0)	14 (3.5)	7 (1.8)

Total (59 items)	1125 (95.3)	21 (1.8)	0 (0)	6 (0.5)	28 (2.4)	55 (4.7)	71 (6.0)	50 (4.2)	23 (1.9)

**Table 6:** Frequency and percentage (%) of agreed judged problematic segments for the Ab-IAP (35-item).

Items	Number of items with no identified problems	Judged problems				Total problems	Struggled	Struggled but answered item correctly	Insufficient information
						
		Comprehension	Retrieval	Judgement	Response				
Impairment (9 items)	174 (96.7)	3 (1.7)	0 (0)	0 (0)	3 (1.7)	6 (3.3)	20 (11.1)	16 (8.9)	6 (3.3)

Activity Limitation (17 items)	328 (96.5)	0 (0)	0 (0)	1 (0.3)	11 (3.2)	12 (3.5)	16 (4.7)	13 (3.8)	8 (2.4)

Participation Restriction (9 items)	177 (98.3)	1 (0.6)	0 (0)	1 (0.6)	1 (0.6)	3 (1.7)	5 (2.8)	5 (2.8)	0 (0)

Total (35 items)	679 (97)	4 (0.6)	0 (0)	2 (0.3)	15 (2.1)	21 (3)	41 (5.9)	34 (4.9)	14 (2.0)

The inter-rater agreement of the independent coding between the two authors yielded an overall kappa value of 0.38 (inter-rater concordance between 89-98% mean 94%), demonstrating fair agreement [19] that is equivalent with other think aloud studies [20].

### Descriptive account of problems identified

The spontaneous contributions participants made during the 'think aloud' task provides an insight into the type and nature of problems people experience when completing the Ab-IAP measures. The qualitative analysis below is used to demonstrate the key issues that were encountered when completing the measures. Verbatim quotations have been used here to illustrate the two broad themes of comprehension and response issues that emerged from the analysis.

#### Comprehension issues

Comprehension issues were judged as any misunderstanding or confusion relating to word or phrase from the measures instructions, items or response options and whether the participant understood the item in the same way intended by the researcher. It is essential that these issues are investigated as if participants interpret items in different ways from each other, comparison between respondents will be flawed.

##### Misread words

The simplest kind of comprehension problem was when participants misread a word in the item. In the following example the participants misreads "showing" as "showering" and by doing so changes the meaning of the item and answers a different question to the one set by the researchers:

C5: How does your joint problem restrict you showing affection?

*P18.A little there because you got to climb over the bath but you know I got a shower in the bath so it would be certainly a little bit there getting your legs over.*

Male aged 70

Although misreading a word is a simple comprehension mistake that anyone can make when answering a self-completion measures, it is a difficult problem to rectify if non-jargon language has been used in the item construction.

##### Incorrect interpretation of wording: Order effect

Participants interpreted some items with an unintended context due to the previous items influencing their judgement. This resulted in some participants answering a different question to the one intended by the researcher, for example, here a participant interprets an item enquiring about difficulties with sitting as specifically about difficultly of sitting in bed, due to the previous item asking about difficulty lying in bed:

B11: What degree of difficulties do you have sitting?

*P8.Oh this is all to do with the bed. I thought I'd answered this but this is from the bed I see. What degree of difficulty do you have sitting? None*

Female aged 61

The order of the items can change the context in which a particular question is asked and influence the interpretation of the item, especially when items are ambiguous [21]. However as this example demonstrates, even seemingly straightforward items can be misinterpreted due to the influence of previous items and therefore suggests that more contextual information may be needed.

##### Abstract concepts

Problems were identified when the items used abstract concepts that left the participant floundering. Participants on occasions reread an item to try and make sense of it and some participants asked for clarification, which due to the concurrent think aloud design the researchers were not able to provide. Participants frequently verbalised several interpretations of the items, leaving the participants to make a guess at the meaning of the item:

C14: How healthy is your physical environment?

*P14.How healthy? Oh that's a difficult one (-) um how healthy is your physical environment. Oh...How do I interpret that? Is that my physical environment in the city I live or in my home or? Um (-) hmmm. That's not a very good question is it [laughs] how healthy is your physical environment. No that doesn't make sense actually. The answers don't make sense to the question [sighs]. Would say I'd have to go midway between and say a moderate amount because there're probably room for improvement everywhere isn't there I would think... Home, everything, the world, the city the (inaudible)*.

Female aged 61.

##### Unfamiliar terms of phrases

Comprehension problems were also encountered when the item contained unfamiliar terms, which again put pressure on the participant to make sense of the item:

B13: What degree of difficulties do you have walking long distances on the flat (> 1/2 mile)?

*P5.Ah severe. Now this is less than or more than half a mile isn't it? Less I well it's severe but it's got to be less than half a mile...Greater is it? Ha well it's severe whichever way round then. Please can you make that more clear please.*

Female aged 69.

The use of abstract and unfamiliar concepts can be avoided, as if the participants have to guess the meaning of a item as there is no way of knowing how accurate their guesses are, unless you have access to their verbalised thoughts, as the 'think aloud' technique provides.

##### Ambiguous items

Some items were seen to be ambiguous leaving the participant' to struggle to answer due to not being provided with sufficient information for the item to be answered. This was seen as a problem when the item was considered to be vague and led some participants to discuss how sensible some of the items were, and left them to have to decide what the most appropriate response would be

C15: How available to you is the information that you need in your day-to-day life?

*P2: (-) I don't understand that question neither. Well I don't really know what it means really so I can't answer it *- [leaves answer blank].

Female aged 53.

B20: What degree of difficulty do you have in lifting?

*P17: (-) how long is a piece of string um (-) you know what are we lifting er yeah I mean it could be anything from picking up a pencil to er to trying to lift a very heavy box um (-) I would say none I've coped with lifting things and carrying things so I'll say none but the question's a bit wide *- [answers none].

Male aged 70.

These problems can be overcome by providing contextual information within the item (such as an object to be lifted).

#### Response issues

Once the participant have interpreted the item, they then have the task of mapping the retrieved or generated information on to one of the pre-specified response options provided [22]. The qualitative analysis provided an insight into a number of issues that made responding to the items problematic for some participants.

##### Co morbidities

One way in which participants were seen to struggle with items was when they were asked to rate their experiences of arthritis in a single joint when they experienced arthritis in multiple joints. This situation posed a dilemma to some participants, as the experiences were not always easy to separate out. As the example below demonstrates, this can lead to participants providing responses that may not reflect their experiences in the joint that is the focus of the study.

A2: How often have you had severe pain from your arthritis?

*P16. Um (-) well I got arthritis in both knees in both hips in in my hand. I would say quite often but it's not the study joint. Um *- [answered quite often]

Female aged 69.

Providing a clearer context to the items, such as reminding participants of the particular joint which is the focus of the study may help reduce the amount of incorrect data.

##### Adaptation to limitations

It is common for people who have a chronic illness or disability to adapt to their physical limitations and find alternative ways of achieving certain tasks. These adaptations can lead to the individual recalibrating their judgments about severity of their limitations. This change to individual's personal conceptions of their limitations is referred to as 'response shift' [23] and can make longitudinal comparisons problematic due to not knowing if the individual's limitations have improved, or if they have made adaptations. In the examples below individuals provided contextual information that suggests that they have problems achieving the tasks. An external observer may have rated these individuals as having more severe problems with carrying out the tasks than the participants self-assessment, however the contextual information that they have provided suggests that their judgments reflects adaptations they have made in their daily lives.

B23: Do you use a walking stick?

*P9: They gave me a walking stick I was very naughty and I never used it. I've got crutches now and I'm not much better with the crutches to be honest but it's it's very difficult for me having a young baby because if you are trying to carry her it's impossible um, so in my personal case (-) it's difficult because I know I would have to tick occasionally because I do only use them at the moment occasionally. But if you want to know how bad I am how often I should be using walking sticks should be all the time so that's not really going to give the correct information to somebody reading this. Um because I would have to tick occasionally because that is how I do do it. But I've been naughty - *[answers occasionally].

Female aged 32.

B9: What degree of difficulty do you have taking off socks stockings?

*P20. Well I'll take that one off easy and if there's anybody I get them to take that one off [laughs] - *[answers mild].

Female aged 75.

B5: What degree of difficulty do you have in bending to floor?

*P18. Er mild. As I said you got to be at a certain angle you got to put either one of your legs back so you can get down. And when you - especially if you're kneeling down you got a job to get back up [laughs] you got to have - I got one of those kneelers - *[answers mild].

Male aged 70.

##### Implicit notions of stability in individual's comprehension of health

The instructions to the Ab-IAP measures asked participants to respond to the items regarding their experiences in the last four weeks. Participants were asked to choose just one response option, but for some participants this was problematic, since their experience varied depending on the context and their circumstance. This left the participants to choose how to represent their experiences, either by picking their experiences in a certain context, averaging their experiences or as the examples below illustrate, some participants did not follow the measures instructions and chose two response options.

B7: What degree of difficulty do you have putting on socks or stockings?

*P16: (-) if I'm standing extreme if I'm sitting none [laughs] um I'm going to - can I write underneath? I'm going to put none when sitting but I'm not a stork extreme when standing I cannot. I can't put my knickers on or anything when I'm standing I have to sit down *- [answers both none and extreme].

Female aged 69.

A12: How severe is your stiffness after first waking in the morning?

*P5: I haven't got, it's not stiff. How severe is your stiffness after waking in the morning? I'm going to put moderate and severe here because some days are worse than others *- [answers both moderate and severe].

Female aged 69.

##### Normative assumptions

Another type of response problem identified was when the measures set a normative level of activity that may have been beyond the capacity of some of the participants, or when an item asked about an activity that was not applicable to the participant. Some participants discussed the issue at length, trying to make the item relevant to their lives and gave the most appropriate response. Others found it difficult to choose an appropriate response and left the item blank.

B15: What degree of difficulty do you have climbing up and down several flights of stairs?

*P10: It's difficult for me to answer that because I don't have several flights of stairs on a regular occurrence. It's something I rarely do. When would I do that? Probably in a shop. But they have escalators (-) yes it could be ladders couldn't it? Climbing up and down several flights of stairs or steps when I'm decorating *- [answers mild].

Male aged 74.

B13: What degree of difficulty do you have walking long distances on flat half a mile?

*P.13: [laughs] I don't. No I mean I don't walk [laughs]...So what do I put?- *[leaves blank]

Female aged 80.

##### Conceptual issues

Other participants struggled with the conceptual basis of items regarding pain. A problem that is common in self-assessments of pain is asking participants to map their subjective experiences of pain into a fixed response option, when pain is a complex, multidimensional and dynamic event [24-26]. Some participants found it difficult to translate their subjective experiences of pain in the response options provided.

A1: How would you describe the pain you usually have from your joint?

P13: As I say I really cannot explain pain, how do you explain pain? Its awfully difficult...How would you describe the pain you usually have from your joint? (-) I simply don't know how to answer it. I can't say it's extreme because I don't know what (-) I don't know. It's not been mild, it's not moderate, I wouldn't say it's severe - well it is severe to me - [answer left blank].

Female aged 80.

The problems demonstrated here are common to many measures that attempt to gain a simple rating of complex pain experiences [24]. By providing more context to the items, such as the experience of pain in certain circumstances, may make the task easier for participants and reduce the amount of incorrect or missing data. However, further research is needed to explore how participants make assessment of their subjective experiences.

## Discussion

The 'think aloud' analysis indicated that the Ab-IAP measures had few problems. As a result, the Ab-IAP offers uncontaminated measures of the three theoretical constructs i.e. the health components of the ICF, that are interpreted appropriately by respondents. The 'think aloud' analysis on the pool of 59 items identified more problems than in the 35 items in the Ab-IAP. Items were identified that had also been shown to be statistically problematic from previous item analyses [3]. Only 4 items from the pool of 59 items with more than one problem were not removed by the statistical item analysis. Thus statistical methods (for example estimates of internal consistency, factor structure, information/discrimination of items) have been useful in detecting items subsequently found in the 'think aloud' study to be problematic for respondents. These findings that the statistical and the 'think aloud' methods complement each other warrant further study. This think aloud study has informed how a number of items that can be modified to reduce problems in future revisions of the Ab-IAP. The think aloud study has therefore both highlighted which items are problematic and demonstrated the nature of those problems.

When constructing a self-assessment measures, researchers face the dilemma of not producing items that are too wordy - which may create too much response burden, and reduce response rates - but nevertheless contain all the information necessary for items to be comprehended and answered. Ambiguous items can create problems for both participants and researchers as they may be difficult to answer, and the responses generated may not be easy to interpret [9]. Clearly it is best if items are clear, brief and concise, however when the meaning of items are unclear and unspecific this can leave participants floundering as they have to fill in the information not explicitly given in the item. It is therefore important that researchers ensure they provide sufficient contextual information within the item to allow the participant to comprehend and answer the item. More contextual information can be provided to Ab-IAP items that were incorrectly interpreted, due to order effects (e.g. B11 adding chair to 'what degree of difficulties do you have sitting?') or item being ambiguous (e.g. adding an object to be lifted in B20 'what degree of difficulty do you have lifting?').

Other issues that have arisen are not specific to the Ab-IAP but are more fundamental problems with all health outcome measures due to the subjective evaluation of one's health being dynamic and complex. Issues of 'response shift' can be particularly problematic when evaluating recovery from an intervention as any changes noted may be due to participants adapting to deal with daily life, rather than the efficacy of the intervention or problems with the accuracy of the outcome measures. Further work is needed to investigate the impact of response shift as a clinically important cofounder and the best means of measuring response shift [27,28].

The 'think aloud' task allowed for the identification of problems that may otherwise have gone unnoticed. The issue of participants recalibrating their judgments regarding the severity of their limitations is a common problem with self-completion health outcome measures, as the task of answering the items involves individuals making a self-assessment on their health status. Problems that occur due to assuming stability in health status could only be overcome by providing a more context specific item (however normative assumptions may exclude some individuals) or by allowing participants to state a certain context (but this would prohibit comparison between individuals) or by asking about a shorter time frame (but this may not be an accurate representation of their general health status). As Mallinson suggests, "further research is needed to explore the extent to which variations such as these occur within and across individuals" (page 18) [9]. The issue of items that are not relevant to participants could be addressed by adding a "not appropriate" response option; this may filter out participants responses that do not reflect their day-to-day life. However this may also increase the amount of missing data due to it being seen as an 'easy option' [21] and make it difficult to calculate participants overall score for the measures.

Self-assessment health outcome measures are crucial in evaluating the effectiveness of interventions. The face validity of self-assessment measures is dependent on shared understanding of the measures instructions, items and response options [29]. The 'think aloud' technique provides a detailed pre-testing method to investigate how participants understand and interpret self-assessment measures [30]. 'Think aloud' demonstrates a way that qualitative and quantitative methods can compliment each other in developing and refining health outcome measures, taking into account both the distribution and nature of identified problems. The addition of 'struggle' and 'insufficient information' coding categories provided additional information that may not have been obtained using the standard coding categories. It may be of value to revise standard coding schemes to include these categories.

## Limitations

Cognitive interviews are qualitative in nature and so whilst they can indicate problems that are present, they cannot provide quantitative data on the extent or the impact of these problems on survey estimates [31]. The relatively small sample size of 'think aloud' studies does prohibit examination of systematic differences between social groups. However, the present study did purposively sample participants so pre and post-surgery experiences could be explored. A further limitation of the 'think aloud' method is that it relies on participants verbally reporting problems. There are two issues related to this, firstly not all cognitive processes can be verbalised as some happen so quickly, and secondly it is not possible to detect problems that are encountered by participants but not verbalized [4,5]. Despite these limitations, the 'think aloud' method is an effective means of improving how measures are interpreted and answered.

## Conclusions

Participants had minimal difficulties completing the Ab-IAP. Problems were identified in 3.1% of responses in the 35-item measures, This 'think aloud' analysis supported the previously carried out statistical item analysis and illustrated how 'think aloud' methods can compliment traditional statistical methods for item reduction and the use of both methods may advance measurement development. As a result, the new measures are not only theoretically based and psychometrically adequate, it also elicits appropriate responses.

The issue of meaning is absolutely central to understanding subjective views and establishing the face validity of subjective health measures. The 'think aloud' analysis has highlighted many important issues that should be taken into account when constructing questionnaire items for people with osteoarthritis.

## Competing interests

The authors declare that they have no competing interests.

## Authors' contributions

All authors collaborated in designing the study. JH and TM collected the data. JH and BP coded the data. JH performed the analyses, with the assistance of BP and SA. JH and BP drafted the manuscript. All authors read and approved the final manuscript.

## Acknowledgements

The authors would like to thank those who participated in the think aloud interview and to the staff at the orthopaedic clinics that helped with recruitment. This research was funded by the Medical Research Council Health Services Research Collaboration. We thank Professor Paul Dieppe for his support of this work and comments on drafts of the manuscript.

## Pre-publication history

The pre-publication history for this paper can be accessed here:

http://www.biomedcentral.com/1471-2474/11/182/prepub
